# Studio One: A New Teaching Model for Exploring Bio-Inspired Design and Fabrication

**DOI:** 10.3390/biomimetics4020034

**Published:** 2019-04-29

**Authors:** Simon Schleicher, Georgios Kontominas, Tanya Makker, Ioanna Tatli, Yasaman Yavaribajestani

**Affiliations:** Department of Architecture, College of Environmental Design, University of California, Berkeley, 232 Wurster Hall, Berkeley, CA 94720-1800, USA; georgios_kontominas@berkeley.edu (G.K.); tanya_makker@berkeley.edu (T.M.); ioanna_tatli@berkeley.edu (I.T.); yasamanyavari@berkeley.edu (Y.Y.)

**Keywords:** architectural education, bio-inspiration, biomimetics, structures, parametric design, structural analysis, fiber composites

## Abstract

The increasing specialization in architecture has clearly left its marks not only on the general profession but also on architectural education. Many universities around the world react to this development by offering primarily conventional and overly discipline-specific courses that often lack bold new concepts. To remedy this situation, the authors propose an alternative teaching model called Studio One, which seeks to facilitate new dynamic links between architecture and other disciplines based on the interplay between fundamental research, design exploration, and practical application. The goal is to develop an interdisciplinary, collaborative design training that encompasses the best that nature has to teach us, realized through the technology that humans have achieved. At the core of this class is the study of biological structures and the development of bio-inspired construction principles for architectural design. Both aspects are rich sources of innovation and can play an important role in the training of future architects and engineers. This paper seeks to provide a coherent progress report. After a brief introduction to the general objectives of Studio One, the authors will specify the methods and 21st century skills that students learned during this class. Relying on four student capstone projects as examples, the paper will then go into more detail on how natural structures can inspire a new design process, in which students abstract basic biomimetic principles and transfer them into the construction of architectural prototypes and pavilions. Finally, the authors conclude by discussing the particular successes and challenges facing this teaching model and identify the key improvements that may give this program an even bigger impact in the future.

## 1. Introduction

Architecture has always had a long-established reputation of being one of the most universal fields of study. This perception partially roots back to the fifteenth century and the era of the medieval master builders who, as a precursor to the modern architect, embodied the unification of different scientific strands like mathematics, engineering, science, and architecture [1]. Even today, architects are still often considered to be the polymaths among the specialists, since their expertise must span a significant number of subject areas. By now, the profession is not only limited to the description of physical structures, such as buildings and urban environments, but encompasses the technicalities of designing those buildings as well as the methods of construction.

Nevertheless, the job profile of the architect has changed drastically over the past centuries and by now has clearly departed from its origins. Today’s profession is characterized by an increasing specialization, which has contributed to a growing separation between what we can imagine and what we can actually construct. This development, however, is of no surprise and in fact can be seen as a logical consequence to the ever-increasing complexity of modern building tasks. Furthermore, the trend towards specialization is also reflected in the academic training of young architects. Most universities around the world are under high pressure to uphold a set of predefined teaching standards and thus focus their efforts on fundamental knowledge rather than looking beyond the horizon. As a result, course offerings are often perceived as conventional or too discipline-specific. While this framework has its benefits and provides students with the necessary set of skills demanded by the profession, it also holds the danger of creating inner-disciplinary silos that may stifle innovation in the long run.

## 2. Studio One Objectives and Program Framework

Studio One is conceptualized as an alternative teaching model to the increasing specialization in the architectural education. The program is positioned at the forefront of an emerging discipline where architecture, engineering, and biology come together. Adopting this focus, Studio One draws inspiration from other successful role models in Europe, such as the University of Stuttgart’s ITECH program or the programs at Architectural Association on Emergent Technologies & Design (EmTech) and the Design Research Laboratory (DRL) in London. Following their pioneering work, Studio One aims to bring this academic strategy to the United States.

**Figure 1:**
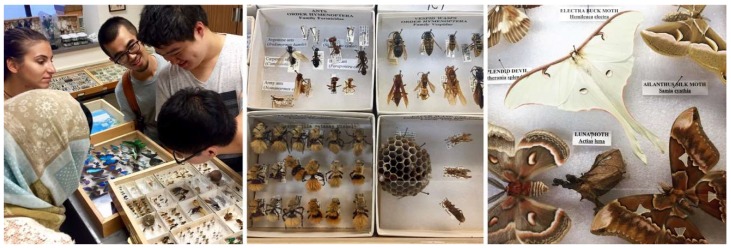
Students on a field trip to UC Berkeley’s Essig Museum of Entomology.

**Figure 2:**
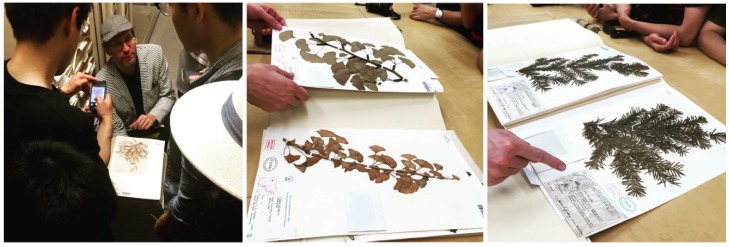
Students study the scientific collection of dried plants at the University and Jepson Herbaria.

**Figure 3:**
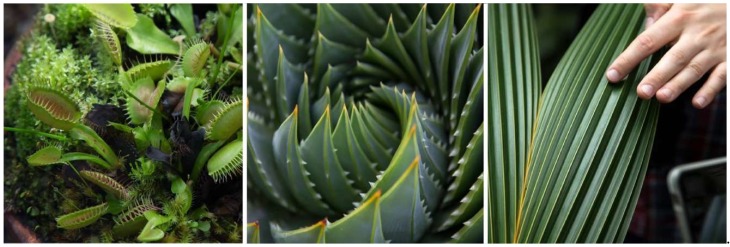
At UC Berkeley’s Botanical Garden, students investigate the morphology of living plants.

**Figure 4:**
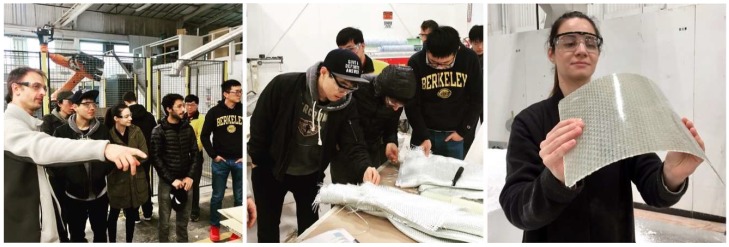
Students learn about composite manufacturing at the workshop of Kreysler & Associates.

Offered at University of California (UC) Berkeley’s Department of Architecture in the College of Environmental Design, Studio One is a one-year post-professional Master’s program for students who already have an accredited Bachelor in Architecture degree and who seek a special, application-oriented, and multidisciplinary learning experience. A two-semester studio class taught by Professor Simon Schleicher makes up the core of the program. It is supplemented by tailored seminars by the Professors Richard Hindle and Marco Cenzatti that support the broader agenda of the program. The typical class size is 10–15 students, for which the university receives applications from around the world. Each year, Studio One seeks to forge new interdisciplinary alliances and long-term industry partnerships. The curriculum itself is designed to mediate between education and research. Aside from gaining basic knowledge within their own fields, the students also gain experience outside of their comfort zones by learning from other disciplines. During the class, a special focus is placed on providing students with the following “21st century skills” as described by the Institute of Museums and Library services: critical thinking and problem solving, creativity and innovation, communication and collaboration, visual and scientific literacy, and cross-disciplinary thinking [2]. To meet these objectives, students are exposed to an inquiry-oriented, experiment-based, and project-driven research agenda. The class is naturally divided into two semesters. In the first semester, students work individually or in small groups. In the second semester, the students join forces and build one project together as a team. Due to the nature of this pedagogical structure, it is possible to push an initial idea beyond the usual scale of small models into larger-scale prototypes and pavilions. To achieve this goal, the class is also supported by a wide network of academic research institutions as well as professionals inside and outside the building industry. Partners at UC Berkeley are, for example, Professor Robert Full and his poly-PEDAL Lab at the Department of Integrative Biology as well as Professor Ronald Fearing and his Biomimetic Millisystems Lab at the Department of Electrical Engineering and Computer Sciences. With its partners, Studio One is contributing to a newly-formed, campus-wide teaching and research initiative called *Design Innovation from Nature*. As part of this initiative, our students collaborated with various museums including the Essig Museum of Entomology, the University and Jepson Herbaria, and the Botanical Garden at UC Berkeley, as shown in Figure 1, Figure 2 and Figure 3. On various field trips, students visited local industry partners such as Autodesk Pier 9, the Otherlab, and Kreysler & Associates, as shown in Figure 4. Over the past two years, Studio One has also found strong support from leading experts and professionals in the field and hosted over 30 guest lectures.

## 3. Research Focus and Methodology

For the academic year 2016–2017 and 2017–2018, Studio One focused on the topic of Bio-inspired Design and Fabrication. This topic was chosen to bring the areas of biomimetics, computation, structural design, and material-based fabrication under one holistic umbrella. With this objective in mind, the class followed an intensive, critical, and analytical approach to cutting-edge design and fabrication methods, while at the same time looking at these aspects from a different angle by studying natural role models. Here in particular, the students challenged the present understanding of architecture through learning how certain construction principles found in plants and insects can improve the efficiency and versatility of buildings and construction processes. Of course, from an architectural point of view, drawing inspiration from nature is not a new concept, but rather a repeating theme in the history of the discipline. However, unlike previous trends that looked into biological forms merely to mimic nature’s appealing aesthetics, the goal for this class is to approach the topic scientifically and to learn from nature’s functional and structural strategies. According to a definition by the Association of German Engineers, “biomimetics combines the disciplines of biology and technology with the goal of solving technical problems through the abstraction, transfer, and application of knowledge gained from biological models” [3]. Furthermore, they explain that “biological models in the sense of this definition are biological processes, materials, structures, functions, organisms, and principles of success as well as the process of evolution itself.” This definition shows that the key idea behind biomimetics or bio-inspiration is not the imitation of natural forms and shapes but the transfer of functional principles to technological applications. The motivation behind this is convincingly simple: over billions of years of evolution, biological structures have found many optimized solutions to a variety of difficult tasks, some of which are quite similar to the challenges technical structures have to meet. Through the constant adaptation to ever-changing environmental conditions, biological organisms have developed such a high-level resilience that some researchers have begun to believe that the real treasure trove of knowledge is the plethora of nature’s successful compromises to often conflicting functional requirements [4].

To implement these biomimetic concepts, the students approached the work in the class from two directions as defined by Speck et al. [5]. The first, called “biology push”, relates to a bottom-up approach. The second is referred to as “technology pull” and describes a top-down process. While the term “biology push” describes a development that is initiated from basic knowledge in biology, “technology pull” refers to the aim of solving a certain technical problem in order to improve an already existing design solution or process. In the context of the class, both directions were investigated simultaneously. Students were given, for example, scientific publications describing promising biological principles or were challenged with a certain problem in architecture for which they had to screen the natural world for inspirational examples.

## 4. Case Studies

To better illustrate the above-mentioned workflow, the following section will present four capstone projects that students developed over the course of the class. The first two are typical examples for a project completed in the first semester, which was done in small teams of two or three. The second two projects showcase a possible outcome at the end of Studio One, when students in the second semester teamed up to work on a larger-scale installation together.

### 4.1. Insect-Inspired Lightweight Facade

#### 4.1.1. Design Brief

The first project prompted the development of a lightweight facade drawing inspiration from the mechanical performance and material properties of rigid insect wings. The two students who envisioned this topic were particularly fascinated by the conflicting functional requirements that flying insects need to address. Insect wings have to be both exceptionally light as well as strong and durable enough to withstand the dynamic loads that occur during flight. This combination of diametrically opposing design constraints promised to be an interesting point of departure.

#### 4.1.2. Biological Role Model

**Figure 5:**
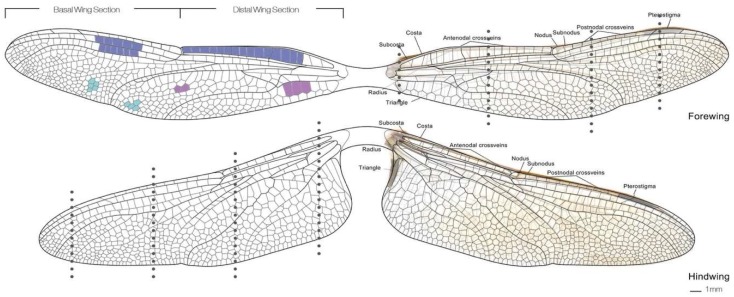
Outlining the vein network in dragonfly wings reveals the distinct variations and local differences in cell geometry and density between the forewing and hindwing of the insect.

In general, rigid and foldable insect wings are a very popular object of investigation since they represent specialized flight organs heavily adapted to the locomotion of individual insects [6,7]. Around the world, researchers are studying insect wings in order to seek inspiration for the design of structurally efficient or deployable systems, as seen in the example of the development of micro air vehicles (MAVs) [8,9]. When the Studio One students looked into this topic, they were particularly intrigued by the work of Wang et al., who investigated the mechanical behavior of dragonfly wings [10]. The common dragonfly (*Anisoptera*) has thin and rigid wings characterized by a very distinctive vein network, as shown in Figure 5. The pattern’s geometry and structure give the wings a special resilience against bending and torsional deflections. Understanding the logic behind the architecture of these vein networks may spark the development of new composite materials and constructions with a high strength-to-weight ratio.

#### 4.1.3. Disclosed Principles and Abstracted Bio-Inspired Structure

**Figure 6:**
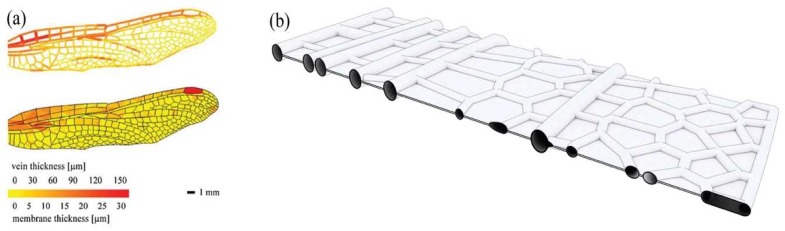
The forewing of the dragonfly features a distinct stiffness distribution (**a**) with thicker veins and membranes shown in red and a sandwich micro-structure shown in cross-section (**b**).

On closer inspection, the venation network in dragonfly wings is composed of a sandwich micro-structure, in which two transparent, membranous surfaces encompass the veins. The veins themselves are filled with hemolymph, a nutrient-containing fluid that gives the wings their flexibility and prevents dehydration. The venation in the fore-and hindwings varies in thickness and cell size, as shown in Figure 6. One can observe different cell forms, ranging from rectangular and hexagonal to other polygonal shapes. Previous research has shown that variations in the network can positively influence the flexural stiffness of the wings [11]. Another remarkable characteristic of the dragonfly wings is their cross-sectional corrugation and undulated geometry of individual cells [12]. Particularly, this cross-sectional corrugation improves the aerodynamics of the wing through the creation of rotating vortices. Inspired by the aforementioned characteristics, the students abstracted the sandwich structure and vein network of the dragonfly wings and transferred these principles into the design of a lightweight facade.

#### 4.1.4. Fabrication and Assembly

**Figure 7:**
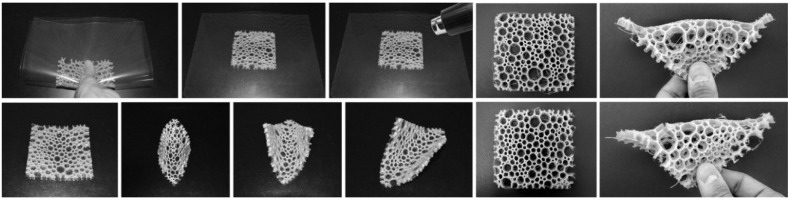
Dragonfly-inspired structure that creates a sandwich effect by wrapping a 3D-printed core made from semi-flex TPU filament between two layers of PETG plastic sheets.

**Figure 8:**
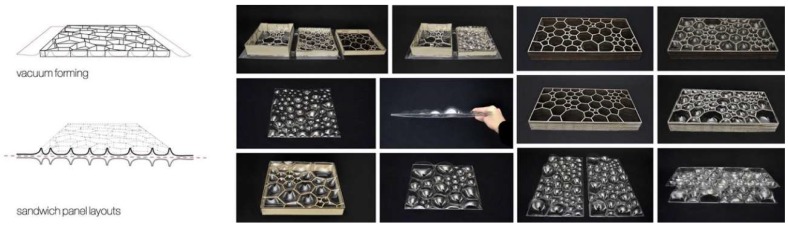
Single and double-layered PETG sandwich panels were fabricated to study the influence of different formwork parameters like materiality, height, and orientation of the mold.

**Figure 9:**
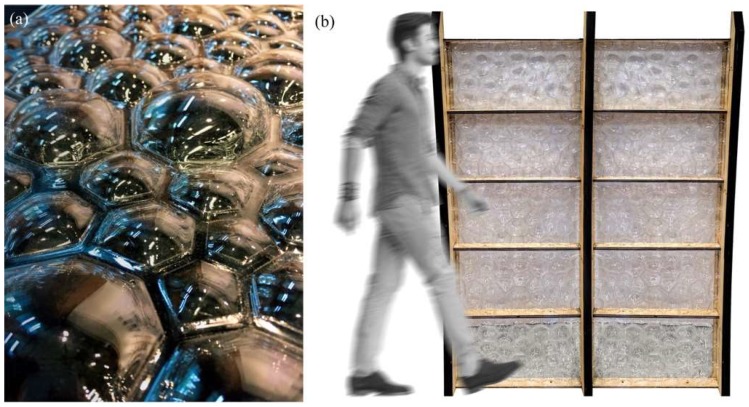
Ten sandwich panels were (**a**) produced by using a sequential vacuum forming process and (**b**) assembled together to form an insect-inspired facade prototype.

In order to investigate the idea of an insect-inspired facade in more detail, the students used a vacuum forming production technique to manufacture a series of models and prototypes in different sizes. Utilizing this technology, the students were able to fabricate multiple low-cost panels from cheap plastic material. In order to meet the requirement of a translucent facade, the team chose 1.5 mm thin sheets of polyethylene terephthalate (PETG) as raw material. To manufacture the wing-inspired venation pattern, the team tested two main techniques. The first technique was based on creating a sandwich structure by thermoforming two layers of plastic around a 3D-printed cellular core, as shown in Figure 7. While this approach resulted in promising and very rigid panels, it had one decisive disadvantage as it was limited to the small bed size of the 3D printer. Thus, to make panels in a larger format, the students followed a different idea. They created the core by laser-cutting thin sheets of plywood and used this structure as a mold to thermoform a first layer of plastic from one side. The corrugated structure was then either directly fused with another plastic layer from the other side, or served as a lost formwork during a second vacuum forming process, as shown in Figure 8. Despite the low stiffness of the individual layers, the sandwich effect created by connecting multiple plastic sheets to each other, gave the final panels a surprisingly high rigidity. To demonstrate the potential of this idea, the students built a facade mock-up, consisting of 10 panels in full scale, as shown in Figure 9.

### 4.2. Plant-Inspired Kinetic Facade Shading System

#### 4.2.1. Design Brief

The second project illustrates another possible direction of Studio One in the first semester. In this study, the students turned to nature to find inspiration for movable systems in architecture. The students expressed interest in kinetic facade shading systems and sought to address challenges in existing mechanical solutions, as they are usually very complex, maintenance intensive, and prone to failure. Seeking a better alternative, the team started their inquiry by conducting an intensive literature research. The team studied publications describing motion principles in plants which have the potential to be translated into low-energy compliant systems without the need for complex mechanical components.

#### 4.2.2. Biological Role Model

Studying plants as concept generators for movable systems might come as a surprise because they are normally considered as sessile organisms that are incapable of any motion. In fact, plant movements usually escape our attention since they are either too slow or too small for the human eye. Yet plants can perform a multitude of different movements that are well-adapted to their environment: some are sudden and vigorous, others delicate and gentle. Their ability to move is particularly astonishing since plants neither have muscles nor a central nervous system. Furthermore, what makes plant movements worth studying is the fact that they often show comprehensive cause-effect mechanisms, in which a certain type of actuation is coupled to a specific functional motion. This is especially apparent in nastic plant movements like flower openings, trapping mechanisms, or pollination principles. Plant organs like leaves and petals, for instance, respond to a triggering stimulus with a movement that is predetermined by their construction. Nastic movements are very common in the plant kingdom and are usually driven by changes of turgor pressure in individual cells and cell clusters, or can also be the result of growth processes.

#### 4.2.3. Disclosed Principles and Abstracted Bio-inspired Structure

**Figure 10:**
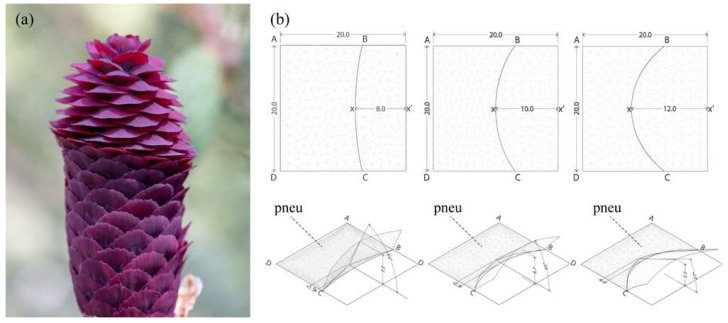
The movable scales of (**a**) the Purple Cone Spruce inspired (**b**) a sensitivity analysis of pneumatically driven curved-line folding models. All models were made using Daniel Piker’s plugin Kangaroo Physics for Rhinoceros 3D by McNeel & Associates. When actuated with the same amount of pressure, models with a lower curvature in the fold responded quicker and showed a larger flapping motion, while models with a higher curvature in the fold were less responsive to actuation and moved significantly slower. (Photo on the left shown with the permission of Jessica Rosenkrantz).

In recent years, more and more research has explored the idea of using nastic plant movements as sources of inspiration for compliant architectural constructions [13]. Prominent examples are the award-winning Flectofin and Flectofold [14,15]. Both of these bio-inspired facade shading systems originated through studying the pollination principle of the Bird-of-Paradise flower (*Strelitzia reginae)* and the snap-tapping mechanism of the Waterwheel Plant (*Aldrovanda vesiculosa)* [16,17]. Common to both systems is a mechanism that couples a bending deformation in one area to a subsequent flapping motion at a different location of the plant organ. In the case of the Flectofin, this effect is due to lateral torsional buckling, as compared to the Flectofold where this behavior is the result of curved-line folding. To further enrich this study, Studio One students brought a third role model into play by looking into the motion principles of conifer cones. The opening and closing movement of the cones are of particular interest as they are based on a bending principle that is driven by passive, hygroscopic swelling and shrinking of a bilayer material [18]. As shown in Figure 10, the students were particularly fascinated by the motion principles in the Purple Cone Spruce (*Picea purpurea*), which combines cell expansion with curved-line folding to allow its seed scales to snap from a positive to a negatively curved geometry.

#### 4.2.4. Fabrication and Assembly

**Figure 11:**
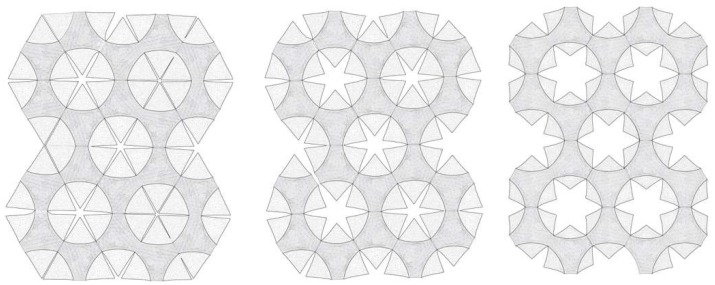
The curved-line folding mechanism can be applied to various patterns and tessellations.

**Figure 12:**
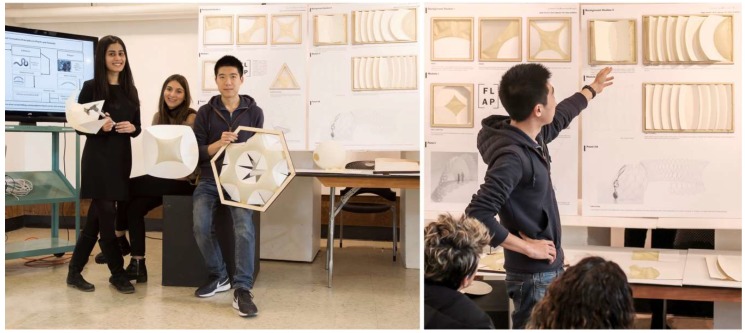
The students learned about the opportunities and limitations of different kinetic systems in various geometrical constellations through building functional models.

To further explore the challenges and opportunities inherent in this precedent, the students integrated the above-mentioned principles into the design of louver-like shading systems and tested them in various geometrical constellations. In this context, choosing a pneumatic actuation turned out to be the most versatile approach as it allowed the students to quickly attach different kinetic models to the same actuator and directly compare the resulting motions. The overarching goal of these experiments was to develop an effective setup to transform small changes in air pressure into an amplified opening movement of the louvers. With this objective in mind, the students focused their efforts on three aspects: (1) fine-tuning the geometry of the curved-line fold in order to improve the sensibility of the flapping louvers in relationship to the inflating cushion; (2) examining whether one pneumatic actuator could drive the movement of multiple louvers; and (3) whether these shading systems could be tightly packed to cover an entire facade, as shown in Figure 11. Students consulted both digital simulations and physical models while investigating these questions. The digital simulations were done using the plugin Grasshopper for Rhinoceros and Kangaroo Physics. These tools were helpful to understand how the main geometrical parameters affected the flapping mechanism. Working with physical models in this project, however, prompted more significant learning as it showed the students the difficulty of fabricating kinetic systems in reality, as seen in Figure 12. These models were built by gluing thin plastic flaps to custom-made latex cushions and inflating them with an air compressor.

### 4.3. Studio One Research Pavilion 2017

#### 4.3.1. Design Brief

The third case study demonstrates a final project of Studio One that was completed in the second semester. For this project, all students along with the instructor joined forces to jointly design and build a research pavilion. The brief was to conceptualize a new temporary (fiber-)glasshouse for UC Berkeley’s Botanical Garden. The project had to be a lightweight and energy-efficient growing environment that could host a selection of the garden’s world-renowned carnivorous plants. In addition, the pavilion had to represent a glasshouse design for the 21st century in step with the latest innovations in the field of digital design and composite manufacturing. Finally, as in the previous projects, this research pavilion had to be informed by design innovations observed in natural structures.

#### 4.3.2. Biological Role Model

**Figure 13:**
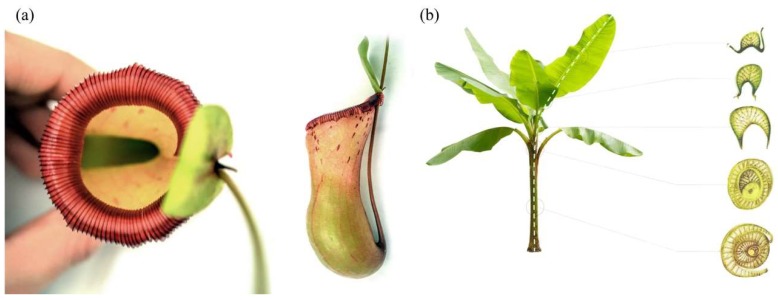
Morphological study of the Pitcher Plant (**a**) and bananas leaf stalks (**b**).

Once again, plants acted as a source of inspiration during the early stages of the design process. This time, however, the team was particularly interested in learning basic strategies for lightweight design and material efficiency. Special focus was placed on two biological role models: the pitcher plant and the stem of banana leaves. The term pitcher plant refers to several carnivorous plants that feature characteristic pitfall traps, as shown in Figure 13a. With these trapping mechanisms, the plants can augment their nutrient uptake from the soil by attracting, killing, and digesting invertebrate prey. Plants in the family of *Nepenthaceae* and *Sarraceniaceae*, for example, have cup-like leaves that are filled with digestive liquid. Additional features like visual lures and nectar bribes are used to attract insects, while slippery surfaces and downward pointing hairs cause the insect to fall inside and prevent their escape [19]. In the context of the class, the team was especially intrigued interested in the pitcher’s rigidity. Even though it is only composed of a very thin cell layer, the cup seems to gain its stability through high global and local curvature. In addition to the mentioned formal inspiration, this general principle was pursued further during the design of the pavilion.

As a second reference, the team studied the functional morphology of banana plants like *Musa acuminata* and *Ensete ventricosum*. As discussed by Ahlquist et al., these plants illustrate a continuous fibrous structure spanning from the tip of their leaves to the network of their roots [20]. Despite their significant height of eight meters, these plants can achieve an astonishing structural capacity and resist enormous wind loads. This impressive performance is largely due to the structure of their stems, as seen in Figure 13b. Looking at a cross-section reveals that the stem is not solid but composed of multiple hollow leaf stalks. The stalks, in turn, are u-shaped structures that consist of two curved shells and a core of connecting lamellas. Several researchers believe it is this curved geometry, in combination with the sandwich-like composition, which give the plant its impressive load bearing capacity. According to recent studies, this construction effectively decreases the forces that act on the stalk by converting bending loads that act on the outer shells into tensile loads within the lamellas, thus reducing the risk of buckling [21,22].

#### 4.3.3. Disclosed Principles and Abstracted Bio-Inspired Structure

**Figure 14:**
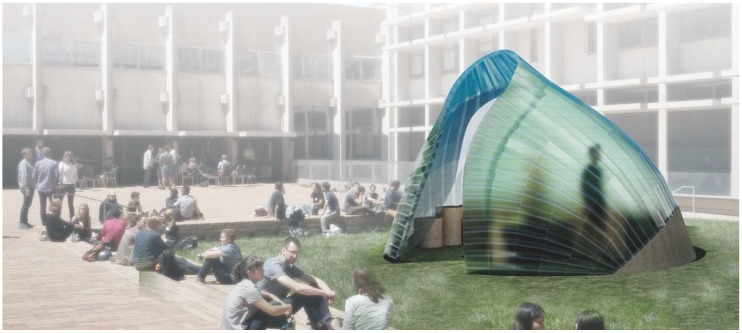
Rendering of the Studio One Research Pavilion 2017 in the courtyard of the College of Environmental Design (CED) at the University of California, Berkeley.

**Figure 15:**
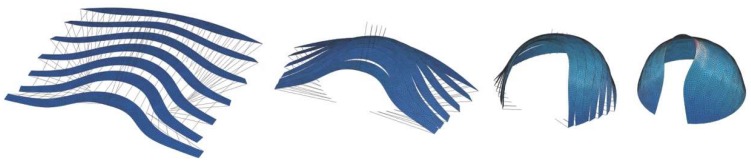
Form-finding of the shell using FE-simulations to ascertain the bent geometry and stresses.

**Figure 16:**
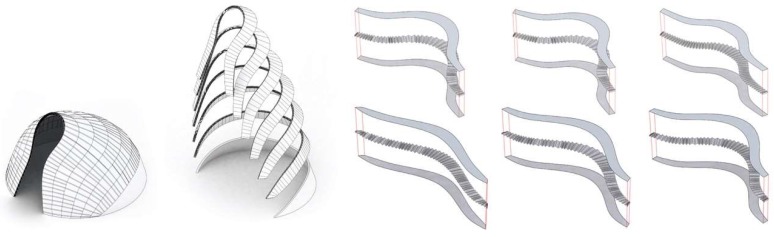
Form-conversion into a multi-layered sandwich shell made from developable surfaces.

**Figure 17:**
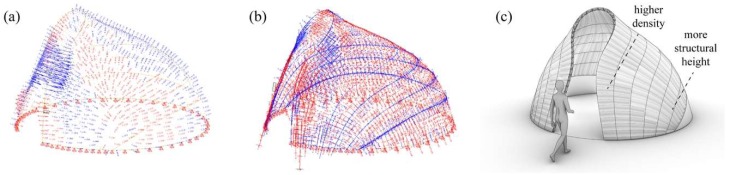
Analyzing (**a**) the deflections and (**b**) stress distribution of the shell informs (**c**) the design of the sandwich structure and affects parameters like the corrugation density and structural height.

Inspired by the intricate geometry of the pitcher plant and the effective buildup of the banana leaf stalk, the students designed the first Studio One Research Pavilion. Figure 14 shows the envisioned structure consisting of a segmented shell made from curved sandwich strips. The proposal builds upon previous research in the area of bending-active structures and pushes this concept one step further. The basic idea behind this design approach is to use large elastic deformations and the bending of initially straight or planar building elements for the construction of curved, load-bearing structures [23,24,25]. However, one of the major challenges for this construction technique is to predict and control the equilibrium shape of multiple bent and interconnected members and to feed this information back into the fabrication process. Another dilemma is even more fundamental and relates to the conflicting goal of building a strong and rigid structure from soft and flexible parts. It is precisely the context of these research questions, in which the students wanted to make a contribution. They designed a bending-active shell by using a series of form-finding and form-conversion techniques as described in more detail in a previous publication by the authors [26]. With the help of digital tools like Kangaroo Physics and Finite Element Methods, the team was able to ascertain the complex curved geometry of the bent strips and analyzed the pavilion’s structural performance as a single-layered shell, as illustrated in Figure 15, Figure 16 and Figure 17. Since a structure made from a single layer of millimeter-thin fiber composite would be far too soft, the team converted each strip into a three-layered sandwich, similarly to the banana leaf stalk. This bio-inspired idea provided the shell with a much higher weight-specific bending stiffness and enabled it to easily carry operational loads and withstand out-of-plane buckling. Additional improvements were made by adjusting other parameters like the height of the sandwich or the density of the corrugation based on the shell’s anticipated displacements, principal shear forces, and direction of principal stresses. While these measures immediately improved the structural capacity of the pavilion, they also came at a high price. The increased rigidity of the sandwich made it impossible to bend the strips in the first place and thus quickly cancelled out the positive effect of the bending-active construction altogether. In response to these challenges, the students proposed another idea that could significantly change future approaches to bending-active structures. Instead of forcing a flat sandwich strip into a curved form, the students bent each layer of the assembly separately and only cross-connected them to each other once the desired shape was reached.

#### 4.3.4. Fabrication and Assembly

**Figure 18:**
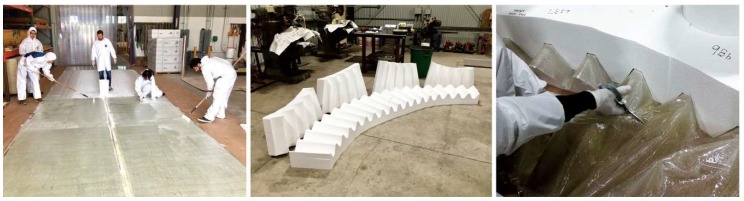
The sandwich core is made from a thin layer of glass-fiber reinforced plastic (GFRP) and pressed into form using a Styrofoam mold that was custom-made on a 4-axis hotwire machine.

**Figure 19:**
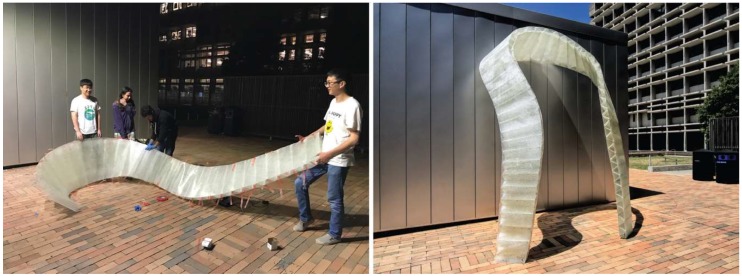
Students assemble the 8-m-long, curved sandwich strip of the pavilion in full scale.

An opportunity to test the practicality of the design emerged through a collaboration with the industry partner Kreysler & Associates. The goal was to construct one strip of the pavilion in full scale. The prototype was built with glass-fiber reinforced plastic (GFRP) and made through a combination of flat-based and minimal formwork lamination, as shown in Figure 18. The laminate was only 1.5 mm thick and composed of a single layer of woven fiberglass with a weight of 600 g/m². During the lamination process, the team used an unsaturated polyester resin that cured at room temperature and did not require any post-processing. A Mylar film was placed on the ground before adding the fiberglass on top, to laminate the upper and lower surfaces of the sandwich directly on the floor. The foil prevented undesired bonding and made it easy to remove the fiberglass after curing. The Mylar film also played an important role during the fabrication of the sandwich core. Once again, the corrugation was laminated flat and covered with Mylar from both sides. Before the layup was fully cured, however, the wet laminate was removed from the floor and placed in between a Styrofoam formwork, which acted as a press. This formwork was cut on a 4-axis hotwire machine, which enabled the production of a semi-flat version of the sandwich corrugation. After placing the wet laminate into the formwork, the superior slip characteristics of the Mylar allowed the fiberglass to easily drape and conform to the shape of the corrugation without sticking to the foam blocks. After curing, all necessary shapes were precisely cut and marked with points for alignment. To assemble the sandwich strip, the students bent each layer separately and then fastened them together through a combination of riveting and gluing. Rivets were used to quickly align and connect the pieces in the correct geometry. Gluing with an industrial double-sided tape created the necessary bond between the sandwich layers and allowed for a transmission of shear, compression, and tensile forces in the structure. Figure 19 shows that the sequential joining technique proved to be very practical and gave the team the chance to cross-connect one corrugation bay after another and slowly bring the 8 m sandwich strip into the correct shape. With this assembly process, the team successfully managed to build the strip with the most challenging geometry in full scale. This achievement can be seen as a first indicator that bio-inspired sandwich principles can be applied to bending-active structures.

### 4.4. Studio One Research Pavilion 2018

#### 4.4.1. Design Brief

The fourth case study is the most recent project of Studio One and is another example of the students’ achievements during the second semester. The Studio One Research Pavilion in 2018 built upon the knowledge gained from its predecessor. This time, however, the priority was on completing all steps of the biomimetic workflow, from the first biological inspiration to a fully completed pavilion. This project was initiated by UC Berkeley’s Department of Architecture which wanted to showcase a large-scale installation during the school’s final exhibition at the end of the academic year. Despite the tight budget, the students were invited to design a reusable pavilion that could have a spatial impact in one of the main courtyards of the College of Environmental Design (CED) at UC Berkeley. Additionally, a material donation from the industry partner Kreysler & Associates further helped to realize the project. The company supported the students with two rolls of pultruded strips made from carbon fiber reinforced polymer (CFRP).

#### 4.4.2. Biological Role Model

**Figure 20:**
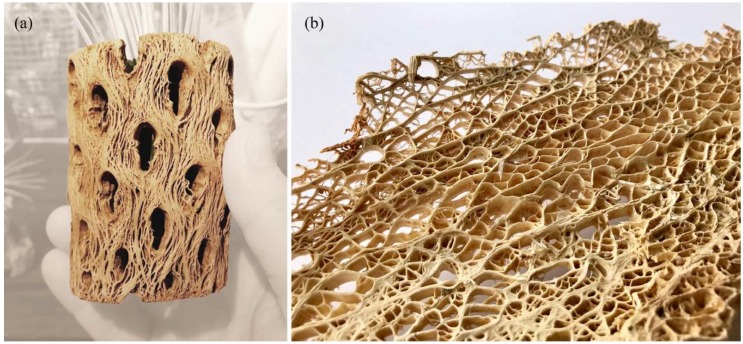
Students study the dried cactus skeletons of the Buckhorn Cholla (**a**) and Prickly Pear (**b**).

Once again, the students were faced with the challenge of finding inspirational role models for the design. Since the construction material of the pavilion, in this case strips of pultruded CFRP, was already known from the start, the team focused its efforts on finding scientific publications and biological samples that demonstrate natural lightweight structures made from fibers. The choice finally fell on wooden skeletons in cacti, specifically the fibrous anatomy in *Cylindropuntia acanthocarpa* (commonly known as Buckhorn Cholla) and *Opuntia basilaris* (known as Prickly Pear), as shown in Figure 20. Unlike smaller, round or barrel-shaped cacti that only consist of water-storing tissue, these cacti can grow much taller and effectively combine water storage with structural support by means of an inner lignified scaffold. In fact, inside the cylindrical stems of the Cholla or the flattened pads of the Prickly Pear, one can find reticulate vascular bundles that form lightweight yet astonishingly strong lattice structures [27,28]. In recent years, these skeletons have fascinated botanists and biomechanics alike. Their research investigates how the cacti’s shape and composition may be the result of specific growth processes and load adaptations that take place on different hierarchical levels [29,30].

#### 4.4.3. Disclosed Principles and Abstracted Bio-Inspired Structure

**Figure 21:**
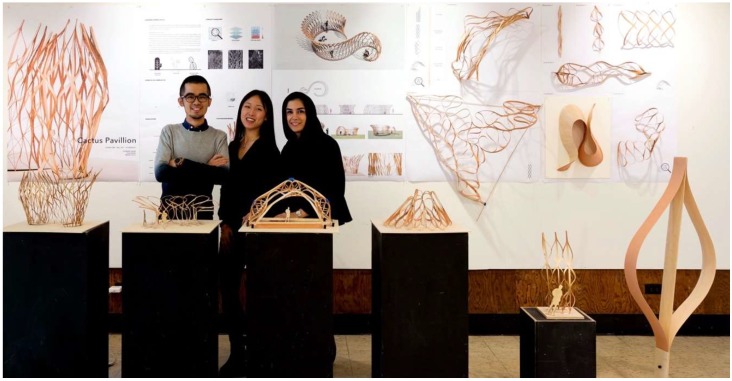
Students explore the design space of lattice structures with a series of physical models.

**Figure 22:**
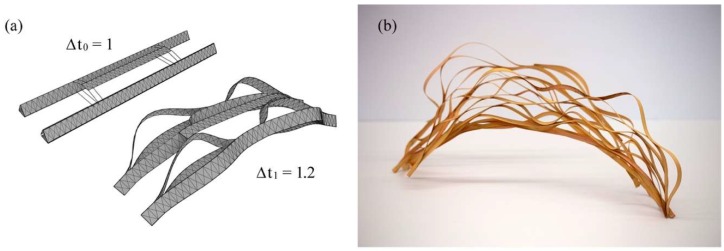
Students use digital growth simulations (**a**) to build strip-based plywood models (**b**).

**Figure 23:**
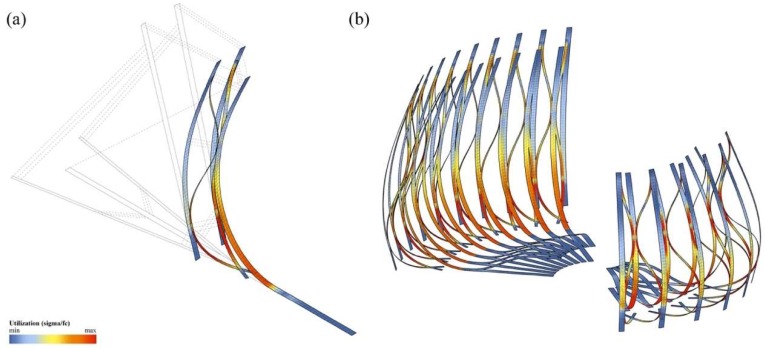
By using advanced FE-simulations the students determined the pavilion’s exact geometry (**a**) and analyzed the stresses in the elastically-bent carbon fiber reinforced strips (**b**).

The students began their own investigations by studying the fiber arrangement and material distribution in dried cactus skeletons. They summarized their observations in a series of design principles, which reflect the plant’s capability to adapt its inner structure in a variety of ways. The first observation relates to the long lateral fibers that seem to have grown in the skeleton without visible discontinuities. This results in an anisotropic structure with a clear directionality and a better force transmission along the lateral axis. The second finding relates to smaller scale undulations and cross-connections of those fibers. The fibers effectively brace the structure and create an interlaced network that is much stiffer than the fibers would be individually. The third observation relates to the distribution of thicker lignified material in the skeleton. By comparing different locations of the skeleton, it was realized that the cactus accumulates more solid material in regions where the anticipated loads are higher than in other parts of the structure. In fact, the skeleton gets visibly thicker and heavier, while also featuring smaller hole sizes closer to the base of the structure. Inspired by these lightweight principles, the students explored multiple paths for their design explorations. On the one hand, they worked extensively with physical models made from thin strips of plywood veneer, as shown in Figure 21. The aim here was to explore the design space created by bending and coupling multiple strips to each other. On the other hand, the team investigated digital means of generating lattice-like structures similar to the cactus skeletons. In this context, it was very helpful to consult previous research by Lienhard et al. and Nabaei et al., who both discussed the form-finding of interlaced strip systems [31,32]. In the end, the team settled on two main strategies to digitally create their designs. The first approach was based on the Rhinoceros plugin Kangaroo Physics, which allows for real-time simulations of particle-spring systems. This interactive tool was very helpful to quickly produce multiple design iterations in the early stages of the creative process. As can be seen in Figure 22, some of these studies began with a densely-packed bundle of interconnected strips that were exposed to different growth rates, which resulted in delamination and a global bending of the entire structure. While this modeling technique created very pleasing visual results and came close to the actual growth processes in the cactus, it was very difficult to steer the development of form. The second technique, therefore, is an attempt to gain more control over the process by implementing Finite Element Methods (FEM) in SOFiSTiK. While this engineering software is typically used for post-design analysis, the students treated it as a design tool to simulate the bending and coupling of multiple strips while observing the increasing stresses in the deflected members, as illustrated in Figure 23. To pull different strips towards each other, the team used the method of contracting cables as described in more detail by Lienhard et al. [32]. This method gradually shortens cable elements between multiple meshes to produce a controlled deformation of the structure without requiring the information of an explicit nodal displacement path. The resulting form-finding process was additionally monitored by a self-developed plugin that checked whether the occurring bending and twisting deformations comply with predefined material limitations and safety factors that were derived from physical experiments.

#### 4.4.4. Fabrication and Assembly

**Figure 24:**
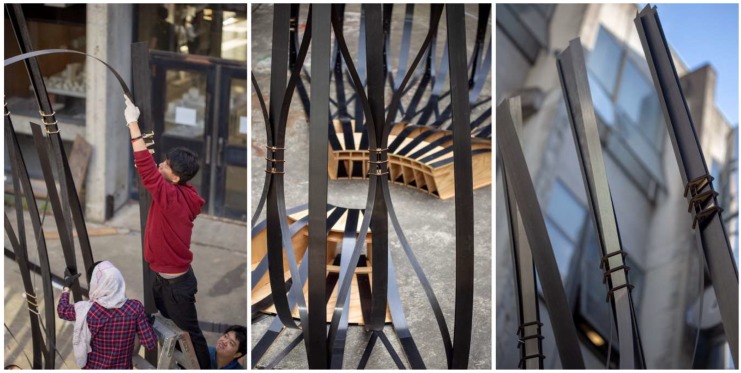
Within two days, the students fabricated and built the 6 m-tall pavilion.

**Figure 25:**
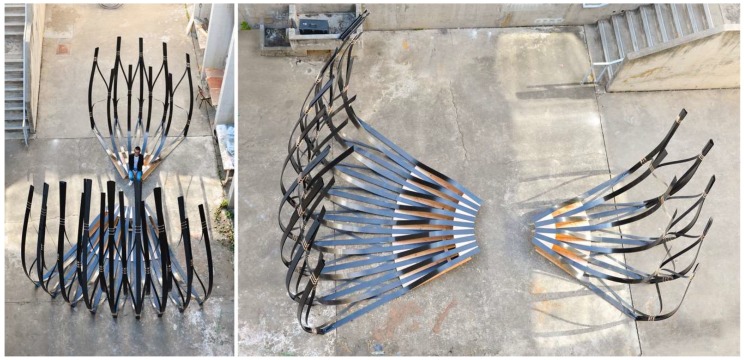
The pavilion as shown during the final exhibition at the end of the semester at UC Berkeley.

In order to test the practical feasibility of the cactus-inspired design strategies, the team designed a pavilion that was made entirely from 1.4 mm thin CFRP strips and sheets of 19 mm thick ACX Plywood. Similar to the biological role model, each strip alone was far too soft to carry bigger compression loads or to reach any notable height. However, when coupled together, the flexible strips supported each other and formed a rigid interlaced system. As shown in Figure 24 and Figure 25, the shape of the pavilion changes from a dense, horizontal bundle of strips to a vertical lattice network, that rises up towards the sky. In fact, the structure reached a height of 6 m with very little material effort. Weighing only 238 kg, this pavilion is extremely lightweight; 178 kg of this total weight was comprised of the bench-like plywood foundation, while the CFRP structure only accounted for 60 kg of the total weight. The students were able to accomplish this impressive result by cross-bracing two types of CFRP strips. The main structure that carried the vertical loads was made from 10 cm wide strips with a total length of 238 m. The secondary structure, which was responsible for stabilizing the system, was made from 5 cm wide strips with a total length of 140 m. Since all strips were fabricated by means of a pultrusion process and thus composed of only a single layer of lateral fibers, they were particularly prone to fracture and needed to be protected against exceedingly high torsional loads and potential local damages. It was therefore important to handle the strips with great care and to follow an assembly sequence that would not deform the strips beyond their material limitations. Furthermore, it was crucial to develop a connection detail that allowed for a quick and easy construction yet was not reliant on any cutting, drilling, or bolting since this would have caused cracks in the strips. In order to address these challenges, the students built the connection details by laser-cutting simple plywood sheets with specific slit layouts that were custom-made for each node and made it easy to insert the strips. This detail simplified the construction process significantly and enabled the students to fabricate and erect the entire pavilion within just two days and to disassemble it after the exhibition in only one day.

## 5. Discussion and Conclusions

Over the past two years through its focus on bio-inspired design and fabrication, Studio One evolved from a vague idea of how architectural education could be improved in the future into a truly profound and meaningful new teaching model. Thanks to newly-established collaborations with various labs and departments on campus, as well as with offices and industry partners across the Bay Area, Studio One has built a strong interdisciplinary network that greatly advances its innovative teaching and research goals. In addition, the increasing popularity and reputation among students, not only within the field of architecture but also beyond, can be seen as proof that Studio One’s inquiry-oriented, experiment-based, and project-driven teaching methodology matches the Zeitgeist and may play an even more important role for the university in the future.

The presented case studies are first indications for the diverse set of skills that students learn in this class and are impressive demonstrations for possible new workflows that reach far beyond disciplinary boundaries. From the perspective of the students, it was an enormous enrichment to push their ideas through different scales from 2D sketches and small 3D models to 1:1 prototypes and pavilions that were built with actual materials and fabrication processes. In preparation for their future professional life, it was crucial that they gained a first-hand experience of working in a larger team. For the personal and professional development of the students, it is extremely important that they learn with and from their peers. It is fundamental for all to understand the difficulties and opportunities inherent in teamwork and to find a way to effectively contribute to a group project.

Of course, Studio One is not free from criticism and clearly has some weaknesses that need to be addressed, but it is set on a good foundation. Nevertheless, the authors see various opportunities for improvements. It would be desirable, for instance, to run the program with exactly the same setup of instruction and thematic focus for a longer period of time. Even though this is not planned at the moment, it would be helpful not only to progressively refine the curriculum but also to establish mutual trust between Studio One and its business and industry collaborators through long-term partnerships. Another key area of improvement would be to diversify the academic background of the participating students. Right now, the admission process for Studio One requires applicants to have a previous degree in architecture. It would be beneficial, however, if the university would widen its criteria and also allow students from other backgrounds to apply. It would be desirable, for example, if students with a previous degree in in biology, product design, engineering, or computer science could join the project team and contribute their unique expertise in the transference of biological role models and bio-inspired principles to new technological tools and processes. Another way to make Studio One even more interdisciplinary without drastically changing the university’s admission process would be to open it up to students that are already in other degree programs like the Master of Architecture (M.Arch.), Master of Science (M.S.), or Doctor in Philosophy (Ph.D.). In fact, over the past two years, many students in other programs and departments signaled their strong interest in Studio One and contributed unofficially to its different projects. Finally, a last aspect that is in dire need for improvement is the access and availability of space and high-end fabrication facilities. Since the program has such a strong focus on hands-on experimentation and digital fabrication, it will be essential to invest in labs with advanced manufacturing capabilities to exploit the full range of both available and future technologies to allow this program to grow and improve in the future. The authors strongly believe, however, that UC Berkeley is uniquely placed to become a major center for this activity and offers a new pedagogical framework that is deeply rooted in both innovative research and cross-disciplinary teaching. Studio One and the supporting initiative of *Design Innovation from Nature* have both been greeted with great success. These developments show that the students and faculty have the right energy and enthusiasm to pursue this trajectory. With proper support, Studio One can draw students, scholars, and practitioners from around the world to develop a built environment that is both beautiful and planet-friendly.
